# The Brain–Skin Connection and the Pathogenesis of Psoriasis: A Review with a Focus on the Serotonergic System

**DOI:** 10.3390/cells9040796

**Published:** 2020-03-26

**Authors:** Ana M. Martins, Andreia Ascenso, Helena M. Ribeiro, Joana Marto

**Affiliations:** Research Institute for Medicines (iMed.ULisboa), Faculdade de Farmácia da Universidade de Lisboa, Av. Professor Gama Pinto, 1649-003 Lisboa, Portugal; amartins@farm-id.pt (A.M.M.); andreiaascenso@ff.ulisboa.pt (A.A.); hribeiro@campus.ul.pt (H.M.R.)

**Keywords:** skin, psoriasis, auto-immunity, inflammation, keratinocytes, cytokines, serotonin

## Abstract

Psoriasis is a common non-communicable chronic immune-mediated skin disease, affecting approximately 125 million people in the world. Its pathogenesis results from a combination of genetic and environmental factors. The pathogenesis of psoriasis seems to be driven by the interaction between innate immune cells, adaptive immune cells and keratinocytes, in a process mediated by cytokines (including interleukins (IL)-6, IL-17 and IL-22, interferon and tumor necrosis factor) and other signaling molecules. This leads to an inflammatory process with increased proliferation of epidermal cells, neo-angiogenesis and infiltration of dendritic cells in the skin. Dysfunctional de novo glucocorticoid synthesis in psoriatic keratinocytes and the skin microbiome have also been suggested as mediators in the pathogenesis of this disease. To understand psoriasis, it is essential to comprehend the processes underlying the skin immunity and neuroendocrinology. This review paper focuses on the skin as a neuroendocrine organ and summarizes what is known about the skin immune system, the brain–skin connection and the role played by the serotonergic system in skin. Subsequently, the alterations of neuroimmune processes and of the serotonergic system in psoriatic skin are discussed, as well as, briefly, the genetic basis of psoriasis.

## 1. Introduction

The skin is an essential organ, the largest of the human body. Being the first barrier that protects the body from the outside environment, it has complex immune, nervous and endocrine systems that allow it to respond in a timely manner to trauma, pathogen invasion, temperature, radiation, allergens and toxins (Section 2.1 and Section 2.2). The complex skin immune system involves innate and adaptive immune cells, skin cells such as keratinocytes (KC) and melanocytes, and an intricate signaling network between all these cellular components that includes cytokines, autoantigens, chemokines, etc. In the last decades, it became increasingly evident that there is a strong brain–skin connection, evidenced by the fact that skin has a fully functional peripheral equivalent of the hypothalamic-pituitary-adrenal axis (HPA) [1] (Section 2.1). Additionally, serotonin (5-hydroxytryptamine; 5-HT), a classical neurotransmitter with a fundamental role in the central nervous system, has been also shown to play an essential role in skin, acting as a mediator between this organ and the neuroendocrine system (Section 2.3). Serotonin is known for the profound effects it has both at the central and peripheral levels of the neuroendocrine system, being involved in the regulation of physiological states and behaviors, e.g., pain, appetite, mood and sleep [2]. Interestingly, there is increasing evidence that the serotonergic system is also a regulator of immune signaling [3]. Serotonin is produced from L-tryptophan, mainly in the periphery, by enterochromaffin cells in the gut and is mainly stored in platelets [4]. It exerts its actions via interactions with different receptors that belong to seven different families, 5-HT1R to 5-HT7R [5]. Equally important is the role of the 5-HT transporter (5-HTT or SERT), which is mainly expressed in platelets and mediates both the release and the uptake of 5-HT [6].

In skin, 5-HT is involved in processes such as vasodilation, inflammation, immunomodulation and pruritogenic effects [7,8,9]. It has been shown that several skin cells, immunocytes and non-immunocytes, can produce and/or metabolize 5-HT and can express SERT and/or 5-HT receptors, emphasizing the important role of this molecule in skin homeostasis. Serotonin, along with stress mediators, also contributes to the effects of psychological stress on the disruption of skin homeostasis [8]. It is widely known that stress can aggravate cutaneous diseases, such as psoriasis and atopic dermatitis [10], that it can delay wound healing and that it contributes to recurring viral infections [11] (Section 2.4).

This review focuses on psoriasis, one of the skin diseases that can be aggravated by and, reversely, can cause psychological stress. Psoriasis is a dermatological disease characterized by red, scaly, well-demarcated plaques on the skin. Although there are several subtypes of psoriasis, it is currently accepted that the disease has genetic, inflammatory and immune components [12] (Section 3.1 and Section 3.2). The main triggers of psoriasis include trauma, systemic use of β-blockers, antimalarials, non-steroidal anti-inflammatories, some infections and psychological stress [13]. It is well known that psoriasis is both affected by stress and leads to stress, possibly causing depression and anxiety which, in turn, exacerbate psoriasis.

## 2. The Brain–Skin Connection

### 2.1. The Skin as a Neuroendocrine Organ

Skin is a vital peripheral organ, the largest of the human body. It possesses a complex structure, being organized in three structural layers: the epidermis, the dermis and the deeper subcutaneous tissue, hypodermis, which is made of fat and connective tissue [14].

The epidermis is the most superficial and biologically active of the three layers; the maturation of the major epidermal cells, the KCs, subdivides the epidermis into four different layers, from the innermost to the outermost: *stratum basale, stratum spinosum, stratum granulosum* and *stratum corneum* [14]. The synthesis of cells and their continuous movement toward the outer skin layers are responsible for the dynamic character of this important organ. Indeed, the skin turnover relates to the transformation of basal KCs into anucleate corneocytes, over a period of approximately 50 days in healthy skin [15]. The *stratum basale* contains KCs, melanocytes and Merkel cells, highly specialized cells which have contact with many axon terminals [8]. The *stratum spinosum* contains Langerhans cells (LCs) which have a fundamental role in skin immunity [16] (Section 2.2.). The *stratum corneum* (SC) is the outmost layer of the epidermis and it has a central barrier role. In the SC the KCs are flattened and denucleated, turning into corneocytes which are surrounded by cornified envelopes that substitute the cell membranes. The intercellular spaces are filled with lipids that help build a very hydrophobic barrier. The SC is not continuous because of skin appendages such as hair follicles and sweat ducts; in these appendages tight junctions provide the primary barrier. In these areas there are live KCs coexisting with the skin microbiota, numerous microorganisms that live commensally and that may affect the immune reactions of the skin [16]. The dermis, which is separated from the epidermis by a continuous basement membrane, is composed of two distinct areas: a superficial thin papillary layer and a deeper thick reticular layer, which is formed from distinct fibroblasts that are responsible for the production of elastin, collagens and structural proteoglycans, forming an abundant extracellular matrix that is used as a scaffold for immune cell migration [16]. Blood and lymphatic vessels are also widely distributed throughout the dermis, forming an important network through which immune cells enter from the blood and exit to the lymphatic nodes [17]. Finally, the hypodermis is a thick layer mainly consisting of loose connective tissue, where blood vessels, nervous cells and fibers are located, and whose main functions are the insulation and protection of the underneath tissues and organs [14].

Skin’s primary function is a protective one, to prevent desiccation and to act as a barrier to physical, chemical and biological environmental agents, separating the external environment from the internal homeostasis [18]. The skin also has a well-characterized exocrine function, performed by sweat and sebaceous glands and hair follicles, which strengthen the epidermal barrier, are involved in thermoregulation, have a protection function against microorganisms, and are also involved in mating via the secretion of pheromones [18]. Adding to its protective and exocrine functions, the skin also possesses metabolic and endocrine competences [18]. The neuroendocrine function of the skin relies on its capacity to communicate with the central neuroendocrine system and to regulate local homeostasis, by producing and/or releasing hormones, neuropeptides, neurotransmitters and other bioregulators [9]. Communication with the central neuroendocrine system is possible due to the high vascularization and the several nerve endings that exist in skin; the biologically active compounds found in the skin are not only locally produced but may be actively transported from blood, released from nerves or from migrating immune cells [18,19] and, reversely, they can diffuse to blood or activate local nerve endings thus influencing central systems such as the brain [18,20]. Importantly, skin cells also have fully functional serotoninergic and melatoninergic systems [19,21]. These functions led to the suggestion that the skin has a fully functional peripheral equivalent of the HPA [10], which is further supported by the fact that the skin and brain share a common embryologic origin, both deriving from ectoblast differentiation, one of the three primary germ layers of the embryo [8].

### 2.2. The Skin Immune System

Most of what is known about skin immunity is based on mouse models. However, although these models have been fundamental to unraveling many processes involved in skin immunity, it must be mentioned that mice skin structure is very different from human skin, with key differences, for example, at the level of innate immune cells and the antigens these present to activate T cells. A recent paper reviewed the use of murine models to understand skin immunity, especially at the level of psoriasis, and stresses the main similarities and differences with human skin [22].

The skin immune system is composed of resident and recruited innate immune system (IIS) and adaptive immune system (AIS) cells that are activated by epidermal structures, microorganisms and other stimuli, and that crosstalk with skin cells, mainly KCs, in order to repair the skin barrier [23,24]. The initiation of immune responses in the skin is due to signals of the IIS, while the cells and signaling molecules of the AIS prolong inflammation [25]. Both innate and adaptive responses employ signaling molecules such as cytokines and chemokines, which are produced by immunocytes or non-immunocytes, and may have a proinflammatory effect (e.g., interleukins (IL)-1, IL-2, IL-12, IL-17, IL-18, tumor necrosis factor (TNF)-α, interferon (IFN)-γ transforming growth factor (TGF)-β1), or an anti-inflammatory one (e.g., IL-4, IL-10) [26].

**Figure 1:**
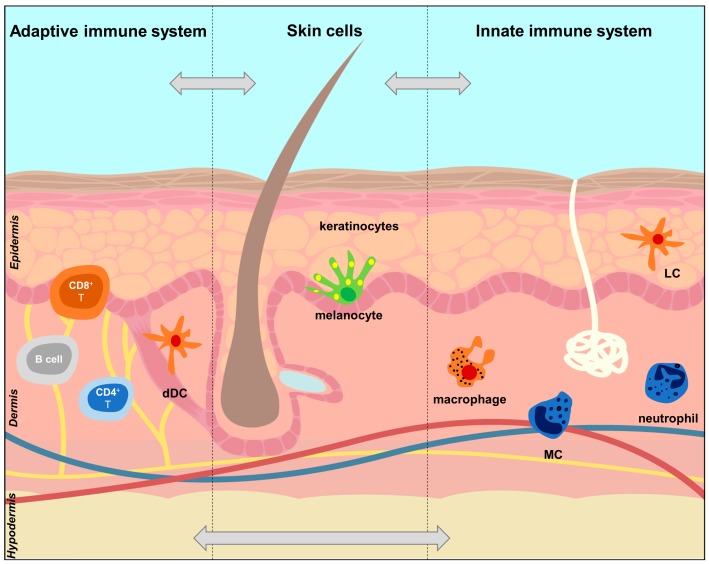
Location of key cells of the innate and adaptive immune systems in the skin. The figure shows the main participants in the innate immune system (IIS) and adaptive immune system (AIS) in the epidermis and dermis. The cells of the IIS have pattern recognition receptors (PPRs) on their surface. Once these are activated, the cells shown in orange (LCs, Langerhans cells, and dDCs, dermal dendritic cells) acquire potent antigen-presenting capacities (APCs) and produce proinflammatory cytokines. The activated orange cells also drive the differentiation of T cells into Treg, Th1, Th2 and Th17 which are part of the AIS [16,25]. Additionally, the APCs also control the influx of neutrophils through the production of TNF and with the help of mast cells (MCs) [25]. Keratinocytes and melanocytes produce autoantigens that are involved in the activation of IIS cells and subsequent differentiation of T cells. Keratinocytes are particularly important in skin immunity, interacting with cells of the IIS and AIS.

The cells involved in skin immunity have different skin locations (Figure 1). In the epidermis, coexisting with KCs and melanocytes, there are LCs, which are dendritic cells (DCs) of the IIS, and the only antigen-presenting cells (APC) present in healthy epidermis, which highlights their role as first line fighters [24,27]. Additionally, CD8+ resident memory T cells of the AIS can also be found in the epidermis. In the dermis, there are several innate immune cells, which include DCs (dDCs), macrophages, mast cells (MCs), and innate lymphoid cells (ILCs). In steady state, the dermal IIS cells survey the dermis for pathogens and, upon inflammation, a much larger number of immune cells concentrate in this skin layer [16] (Section 3.2). Adaptive immune cells present in the dermis include B cells and CD4+ T helper (CD4+ Th) cells [16]. In healthy dermis, CD4+ T and regulatory T cells (Treg) are abundant, while B cells are rare [17]. Additionally, heterogeneous populations of dermal fibroblasts, as well as adipocytes, have a role in skin immunity [16]. It should be kept in mind that mammalian skin has a remarkable architectural heterogeneity, with different epidermal and dermal thickness, densities of hair follicles, sebaceous and sweat glands, microbiome, etc., and this is reflected in the composition of the immune cells in different skin areas. Hair follicles, for example, have their own unique epidermal and dermal immune microcosmos, and their KCs are essential for recruitment and retention of immune cells, in homeostasis or under stress [23].

The initiation of the skin immune response occurs when the skin barrier is broken (by wounds or through tight junctions in the skin appendages that lack the SC). Cutaneous LCs are attracted to the epidermis in a chemotactic process induced by chemokines produced by KCs [24,27] and are able to elongate their dendrites outward beyond the tight junctions to uptake external antigens [16]. Keratinocytes act not only as a physical barrier in the epidermis, but also have sensors that recognize foreign or microbial agents and tissue damage, through pattern recognition receptors which, upon stimulation, lead to the production of secondary mediators, such as antimicrobial peptides (AMPs) and pro-inflammatory chemokines and cytokines. These, in turn, promote AIS responses, particularly of the Th1 and Th17-type [23]. A dysregulation of AMP production seems to be a contributing factor for the development of autoimmune diseases, such as psoriasis [28] (Section 3.2). Dermal DCs also contribute to an efficient cytokine and chemokine network in response to inflammation and activating the AIS response [29]. During inflammation, plasmacytoid DCs (pDCs) are recruited into peripheral locations [30] and are activated by complexes formed between the AMPs produced by KCs, which become autoantigens after complexing self-DNA released from damaged KCs and neutrophils [31]. Upon stimulation, pDCs differentiate into DCs and/or may activate myeloid DCs (mDCs) in an IFN-α-dependent manner [31,32]. Dermal MCs also function in the activation and recruitment of immune-competent cells, being able, for example, to activate KCs and communicate with other cells [24]. Additionally, they can behave like APCs, interact with other (professional) APCs, (e.g., LCs and DCs) via TNF-α and histamine, which is essential for these to migrate, maturate and present the antigen [33].

**Figure 2:**
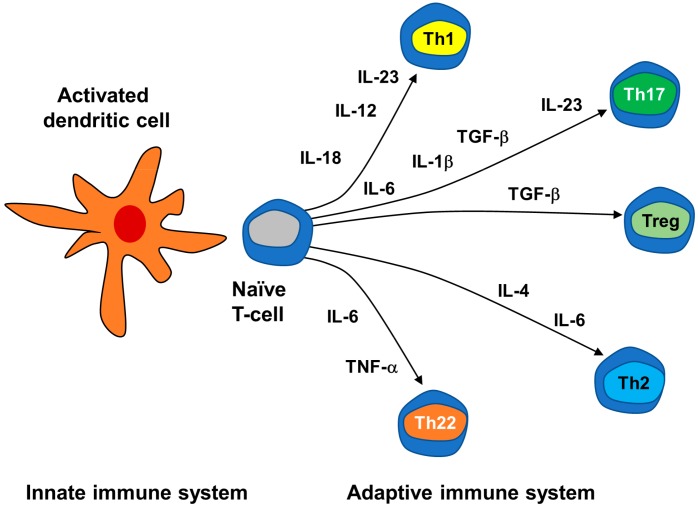
Differentiation of naïve T cells induced by cytokines produced by dendritic cells of the innate immune system.

There is increasing evidence that DCs are the key cells connecting the innate immunity to the adaptive T-cell response [34]: once DCs and other APCs present the antigen, they can activate the skin AIS, mainly represented by T lymphocytes [25]. Activation of APCs through PPRs drives the differentiation of naïve T cells into different functional phenotypes Th1, Th2, Th17, Th22, Treg and others, which are subtypes of CD4+Th cells. Expression of co-stimulatory molecules by the APCs determines if the naïve T cells are ignored, silenced or activated. Then, the production of cytokines such as IL-6, IL-10, IL-12 and IL-23 determine to which phenotype the naïve T cells are to be differentiated [25] (Figure 2).

### 2.3. The Serotonin System and Its Importance in Skin Neuroendocrine and Immune Systems

#### 2.3.1. Serotonin Metabolism, Transport and Function

Serotonin is one of the oldest known neurotransmitters, with important roles in several physiological processes. It is essential for cell proliferation, differentiation, migration and synaptogenesis, regulation of leukocyte chemotaxis, cytokine production and activation of T cells. It is broadly distributed in living organisms, which is evidence of its key role in maintaining homeostasis [35].

Serotonin is a monoamine mainly synthesized from L-tryptophan by enterochromaffin cells in the digestive tract and is stored in platelets [4]. The initial 5-HT biosynthetic reaction, converting L-tryptophan to 5-OH-tryptophan, is catalyzed by the enzyme tryptophan hydroxylase (TPH, encoded by the gene TPH1 or, in the brain, by TPH2) [7]. The 5-OH-tryptophan is then decarboxylated by the L-aromatic amino acid decarboxylase, to produce 5-HT [8]. The main catabolic pathway for 5-HT is its conversion to 5-hydroxy-3-indoloacetaldehyde (5-HIAL), a deamination reaction catalyzed by the outer mitochondrial membrane monoamine oxidase (MAO) [36].

Serotonin actions are mediated through interactions with membrane-bound receptors. There are seven general families of cell surface 5-HT receptors (5-HT1 to 5-HT7), with at least 21 subtypes [5]. There are two types of these receptors: metabotropic receptors are coupled to G proteins and act by stimulating/attenuating adenylate cyclase activity or via the enhancement of phosphoinositol hydrolase activity, while ionotropic receptors act as ion channels [8]. The receptor 5-HT1R acts by attenuating adenylate cyclase, inhibiting inflammation, while 5-HT7R acts by stimulating this enzyme activity; 5-HT2AR acts via the enhancement of phosphoinositol hydrolases activity, promoting inflammation, while 5-HT3R functions as an ion channel [6,8]. The 5-HT transporter, SERT, is also an important component of the serotonergic system. This protein is mainly expressed in platelets, and is involved in both the release and reuptake of 5-HT from these cells [6]. The 5-HT transporter is the target of the well-known specific serotonin reuptake inhibitors (SSRIs), antidepressant drugs that inhibit the reuptake process.

#### 2.3.2. Serotonin and the Immune System

Serotonin is a classical neurotransmitter but several studies have been revealing an important role for this monoamine outside of the central nervous system: 5-HT and its receptors have an important role in the regulation of immune signaling. Almost all circulating 5-HT is stored in platelets and is released following platelet activation in response to damaged endothelium or ischemia [37]. However, the synthesis and transport of 5-HT in immune cells (innate and adaptive) is much more diverse than initially thought, and includes T cells, macrophages, DCs and others (Table 1) [4,38,39].

When studying the effect of 5-HT in the immune system, one must take into account that, although several 5-HT receptors and the SERT have been found in numerous immunocytes and non-immunocytes, some studies were only performed at the mRNA level, which is not evidence for the presence of a functional protein. Moreover, the stimulation of 5-HT receptors is usually made by artificial means, which does not distinguish between cell types. For example, in the case of T cells, the stimulation does not distinguish between CD4+ and CD8+ T cells or their specific subtypes, e.g., Th17 and Treg [39]. Furthermore, some results were obtained with animal models or non-human cells and differences can be expected [35,38]. However, there is evidence that, in both rodent models and humans, 5-HT plays a role in regulating inflammation and immunity, acting as a strong chemoattractant to recruit innate immune cells to inflammation sites, modulating the production of cytokines and chemokines and being involved in cell activation and proliferation [58].

In vitro studies with mice DC and T cells in culture suggested that 5-HT and serotonergic signaling play a role in the connection between IIS and AIS, participating in the cross-talk between DCs and T cells and influencing T cell activation. Dendritic cells are able to take up 5-HT at inflammation sites, via SERT, from the microenvironment and from activated T cells expressing the TPH1 enzyme, shuttling it to naïve T cells, thus modulating T-cell proliferation and differentiation [35]. Analysis of 5-HTR expression in primary mouse T cells further suggested that the 5-HT7R is involved in the activation of naïve T cells by 5-HT, since these cells primarily express this receptor [38].

At the IIS level, 5-HT and its actions have been studied in neutrophils, monocytes, macrophages, eosinophils and MCs [58]. Studies with mouse and human MCs have shown that these cells are capable of synthesizing and storing 5-HT [42], as well as expressing mRNA for several 5-HT receptors [43]. Furthermore, 5-HT induces MC adhesion and migration to inflammation sites, in a process mainly involving the 5-HT1A receptor [43]. Serotonin released by platelets is also able to promote the recruitment of neutrophils in acute inflammation, but the receptor(s) involved is/are still unknown [58]. Dendritic cells express many receptor types (Table 1): while immature DCs mainly express mRNA for 5-HT1B/E and 5-HT2A/B receptors, both immature and mature cells can express 5-HT3, and mature DCs preferentially express 5-HT4 and 5-HT7 receptors [58]. Moreover, 5-HT is able to alter the cytokine production in DCs, increasing IL-1β, a potent proinflammatory cytokine, and IL-8, a chemotactic factor, while decreasing IL-12 and TNF-α [41]. In vitro studies using human monocyte-derived DCs and in vivo studies using mice showed that 5-HT induced migration of immature DCs, but not mature ones, via activation of 5-HT1B and 5-HT2 subtype receptors. Furthermore, 5-HT enhanced the production of IL-6 in mature DCs via 5-HT3R, 5-HT4R and 5-HT7R, and of IL-10 by activating 5-HT4R and 5-HT7R, globally inducing a Th2 polarization of CD4+ T cells [59]. In vitro studies using mice bone marrow-derived DCs showed that the expression of HT7R was upregulated in mature DCs, being involved in the morphology and migratory properties of these cells [60].

At the AIS level, 5-HT is also produced, metabolized and exerts its action in several cells (Table 1). Serotonin is involved in T cell activation, and these cells are able to synthesize 5-HT by T cells, as shown by increased levels of TPH1 expression [35,38]. Interestingly, levels of 5-HT metabolic enzymes TPH1 and MAO are higher in CD8+ T cells than in CD4+ T cells, suggesting a specific role for 5-HT in this cellular subset [48]. Like in DCs, the expression of 5-HTRs in T cells depends on the maturation state: in naïve murine T cells, 5-HT7R is the primary expressed receptor, while upon cell activation there is an upregulation of 5-HT7, 5-HT1B and 5-HT2A receptors [38]. The receptor 5-HT1B was shown to be involved in the proliferation of murine and human CD4+ T cells [47], while 5-HT2AR plays a role in the T-cell receptor mediated IL-2 and INF-γ production [61]. In human cells, 5-HT2BR is the most expressed receptor during CD4+ T cell differentiation [50]. The 5-HT3R, expressed in human but not mouse T cells, seems to be involved in the migration of these cells toward gradients of the cytokine CXCL12 [52]. It was also shown that 5-HT induces the secretion of IL-16 from human CD8+ T cells, possibly via 5-HT2 receptors, and this proinflammatory IL acts as a chemotactic agent for the recruitment of CD4+ T cells into an inflammatory focus [62]. The role of 5-HT in B lymphocytes has also been studied. These cells express SERT, with a marked increased expression upon activation [53], and the uptake of 5-HT by B cells has been shown to cause apoptosis [63]. Serotonin also increases B lymphocytes proliferation via 5-HT1A receptors [54]. The receptor 5-HT3R was also detected, at the protein level, in B cells in germinal phase, but not in naive ones [55].

The interaction between serotonergic and immune systems is bidirectional, thus the actions of the 5-HT system are also regulated by elements of the immune system, such as cytokines. For example, indoleamine 2,3-deoxygenase, the first enzyme of tryptophan catabolism, expressed by macrophages and DCs, is induced by the Th1-type IFN-γ, leading to a decrease in the concentrations of tryptophan and 5-HT and accounting for the antiproliferative effect of IFN-γ [64]. Furthermore, proinflammatory cytokines such as IL-1β can alter the metabolism and release of 5-HT in the CNS via regulation of SERT [65].

#### 2.3.3. Serotonin in Skin

Serotonin, being involved in endocrine and immune functions, also has important roles in skin, acting as a mediator between this organ and the neuroendocrine system at both central and peripheral levels [8,9].

**Figure 3:**
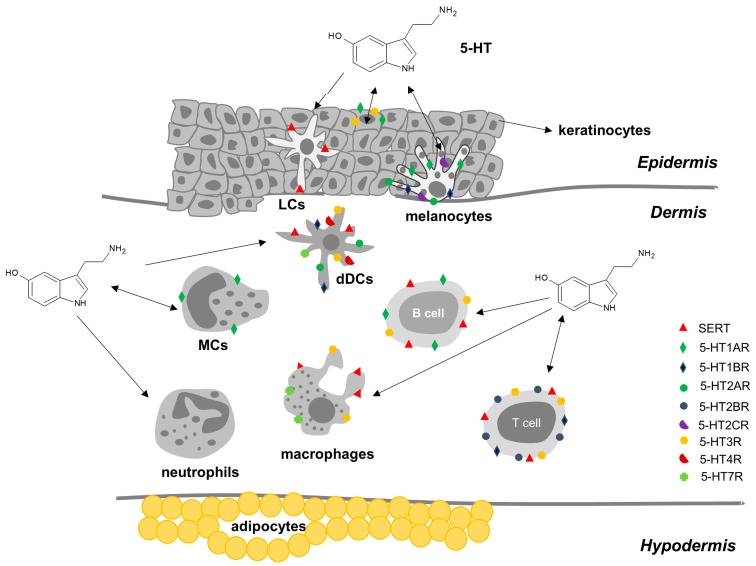
Serotonin, serotonin transporter (SERT) and serotonin receptors (5-HTRs) in human skin immunocytes and non-immunocytes. The cell activation state and environment influence the effects of 5-hydroxytryptamine (5-HT) by changing the receptor and transporter expression. The figure shows all the membrane proteins that may be present at any given time. Double arrows represent the production of 5-HT by the cells and the effect of 5-HT on the same cells; single arrows initiating in cells represent the production of 5-HT by these cells, while arrows initiating in 5-HT represent the direct or indirect effects of 5-HT on these cells. LCs, Langerhans cells; dDCs, dermal dendritic cells; MCs, mast cells.

In skin, 5-HT is involved in vasodilation, inflammation (associated with MCs secretion), and immunomodulation, and also has pruritogenic effects [7]. Several studies (reviewed in [8]) revealed the presence of 5-HT and 5-HT biosynthetic enzymes in several human skin cells (Table 1) including KCs, melanocytes, dermal fibroblasts, MCs, Merkel cells, T cells, natural killer (NK) cells, LCs, sensory nerve endings and release from platelets [8,9] (Figure 3). Molecular biology studies reveal that human skin cells express several 5-HT membrane-bound receptors (Table 1). Messenger RNA for 5-HT1AR, 5-HT1BR, 5HT2AR, 5-HT2BR, 5-HT2CR and 5-HT7R was detected and the expression was cell type-specific and was changed in skin pathologies [56]. Additionally, immunohistochemistry assays in human skin showed the presence of 5-HT1AR in the upper part of the epidermis and in papillary dermal mononuclear cells, 5-HT2AR in the epidermis and 5-HT3R in the basal epidermal skin layer [57]. The 5-HT receptors are also expressed on sensory nerve endings, transmitting changes in skin homeostasis to the brain [21].

The receptor 5-HT1A is mainly expressed in MCs and melanocytes, but also in KCs of the upper epidermis and dermal vasculature. The binding of 5-HT to this receptor is possibly involved in the regulation of the differentiation, life-span and dendricity of several skin cell types, and on the modulation of the skin barrier [8]. Additionally, studies with human and mouse MCs have shown that 5-HT can promote inflammation by binding to 5-HT1AR in these cells and recruiting them to the inflammation site [43]. Studies in rats have also evidenced a role for 5-HT1AR in the regulation of the action of sensory neurons, via 5-HT produced by neighboring Merkel cells [66].

The 5-HT2R family is composed of 3 subtypes, A, B and C. The 5-HT2A receptors are the most studied but 5-HT2CR seems to be predominant in human skin [56]. Receptors 5-HT2AR are present on dermal T lymphocytes and on sensory afferent nerves and their activation may lead to mobility of T lymphocytes and influence nerve transmission, respectively [8]. The mRNA for the subtype 5-HT2BR has also been detected in human skin [56]. Receptors 5-HT2C were detected in human epidermal melanocytes [8] and in murine LCs [67], where they possibly have a role in regulating dendricity.

The receptor 5-HT3R is expressed by human basal KCs and is involved in the proliferation of these cells [8]. Additionally, 5-HT3Rs are also located in sensory nerve endings and possibly mediate the pruritus reaction obtained when 5-HT is iontophoretically applied in human skin [68]. 5-HT7R has been detected in human dermal vasculature [8].

The 5-HT transport protein SERT, although mainly expressed in platelets, is also expressed in skin, in Merkel cells, LCs and T lymphocytes, where it might participate in 5-HT uptake, making these cells more susceptible to apoptosis [8].

### 2.4. Effects of Psychological Stress on the Skin

The discussed similarities and communication between skin and brain help explain the detrimental physiological and functional effects that psychological stress can have on the skin, triggering and affecting the development of dermatological disorders.

In fact, stress has been known to aggravate and help spread cutaneous diseases such as psoriasis, atopic dermatitis, seborrheic eczema, prurigo nodularis, chronic urticaria and alopecia areata [10], as well as delaying wound healing and enhancing recurring infections by herpes simplex and herpes zoster viruses [11]. Skin disorders which are characterized by defects in cutaneous permeability barrier function are usually adversely affected by psychological stress [19]. Psychological stress in humans is responsible for delayed barrier function recovery, increased concentrations of cortisol in plasma (which has a negative impact in wound healing, for example), and activation of several inflammation and immune players (e.g., IL-1β, IL-10, TNF-α) [69]. Additionally, it has been suggested that chronic stress contributes to skin aging [11].

The brain–skin connection explains how inflammatory skin diseases (such as psoriasis) can be aggravated by stress. Skin is both a source and a target of key stress mediators, such as cortisol, cortocotropin-releasing hormone, adrenocorticotropic hormone, prolactin and nerve growth factor [1]. Stress conditions cause changes in the immune system response and in the communications with the nervous and endocrine systems. These changes may be due to a direct action, since almost all immune cells express receptors for one or more stress hormones [11], or an indirect effect since stress regulates the production of cytokines such as ILs, TNF-α and IFN-γ [70]. Skin IIS MCs have a key role in the effects of psychological stress in skin. These cells are activated by stress and start producing stress hormones and inflammatory factors, leading to a vicious cycle of stress-induced inflammatory events [11].

Serotonin also plays a role on the effect of psychological stress on the skin. Stress activates the HPA axis, leading to increased circulating levels of corticosteroids, and these stimulate the synthesis and turnover of 5-HT. Furthermore, stress leads to an acute decrease of 5-HT1AR which is expressed in the outer part of the epidermis, either through a direct action of cortisol on gene expression and/or by feedback inhibition. Changes in 5-HT1AR expression might then modulate the protective function of the epidermis [8].

## 3. Psoriasis

### 3.1. Introduction

Psoriasis is an immune-mediated disease that causes raised, red, scaly patches to appear on the skin. It is one of the most common chronic non-communicable diseases [71], affecting approximately 125 million people globally (2–3% of the world population) [72,73]. Costs associated with psoriasis present a considerable economic burden, with the average annual costs of moderate to severe psoriasis being €10,000 per patient per year [74].

Psoriasis is clinically classified in five subtypes: psoriasis vulgaris or plaque psoriasis, the most common form, affecting 90% of the patients [13]; psoriasis guttate, the second most common form, characterized by small, droplet-shaped lesions that appear on the trunk, arms and legs; pustular psoriasis, a rare clinical subtype of psoriasis where the skin shows diffuse redness and subcorneal pustules [75]; erythrodermic psoriasis, also a rare and severe variant of the disease, characterized by a generalized inflammatory erythema in at least 75% of the skin [76]; and inverse psoriasis or flexural psoriasis, a form of plaque psoriasis that affects inverse/intertriginous/flexural body areas, such as ears, axillae, groin and clefts [77]. The most common form of psoriasis is chronic plaque psoriasis, which is characterized by stable and localized erythematous scaly plaques that are well demarcated from normal skin. These range from very few to a high number covering a large area of the skin. Any skin area can be affected, including the scalp (75–90% of psoriasis patients develop scalp psoriasis) and nails [13].

Currently, it is accepted that psoriasis is a complex inflammatory immune-mediated disease with a genetic component [12], and which can be triggered by internal or external stimuli that initiate the inflammatory process in genetically susceptible individuals. Identified stimuli comprise: mild skin trauma such as scratching, piercings and tattoos [13]; systemic drugs such as β-blockers, antimalarials, non-steroidal anti-inflammatory drugs and antidepressants [78]; streptococcal throat infections, which seem to play a role in the initiation and acute exacerbation of psoriasis, especially psoriasis guttate which mainly affects children and young adults [79]; HIV, which might also trigger psoriasis, with HIV patients developing this disease or experiencing exacerbation of preexisting symptoms [78]; and psychological stress [26].

It is well known that psoriasis is both affected by stress and leads to stress, possibly causing depression and anxiety which, in turn, exacerbate psoriasis. Psoriasis and other skin disorders such as acne, alopecia and atopic and seborrheic dermatitis, have been called psychodermatologic disorders, which emphasize the possible interaction between skin and brain in these diseases [80]. Psoriasis effects go beyond the physical symptoms. The disease affects the patient’s personal life (emotional wellbeing, relationships, sexuality, leisure activities), as well as the relationship with others, such as work, social life and family life. This is easily understood because, unlike other autoimmune diseases which are marked by “invisible inflammation”, psoriasis is a highly visible condition and often patients endure social stigma [72]. Thus, it is not surprising that the use of antidepressants in psoriasis patients is higher than in non-psoriatic individuals [81].

Beside depression and anxiety, other less known comorbidities of psoriasis have been described, such as cardiovascular disease, psoriatic arthritis, sleep apnea, osteoporosis and celiac disease [82].

### 3.2. The Pathogenesis of Psoriasis

The current understanding of the pathogenesis of psoriasis results from both planned and unexpected developments in the fields of genetics, cell and molecular biology, and immunology, and from the history of trial-and-error therapies [26]. Initially, psoriasis was thought to be exclusively caused by a dysfunction of limiting KC proliferation, possibly caused by altered cell cycle kinetics [12,26]. The implication of the immune system was suggested in the late 1970s when a patient receiving cyclosporin A for prevention of organ rejection exhibited an improvement of psoriatic lesions [83]. Additional experiments showed that T lymphocytes were responsible for the psoriatic plaques [84].

The histology of a psoriatic plaque perfectly shows the complexity of the disease: it shows epidermal hyperplasia caused by an abnormal proliferation and differentiation of KCs, dermal inflammatory infiltrates composed of dermal DCs, macrophages, T cells, neutrophils and neovascularization [15,26,85,86]. In psoriatic skin, the transformation from basal KCs to corneocytes occurs in around 5 days, in comparison with the roughly 50-day process in healthy skin [15].

The current model of psoriasis pathogenesis considers that it is the crosstalk between the complex network of skin DCs, T cells (mainly Th17 [87]) and resident KCs that generate inflammatory and immune routes which are responsible for the initiation, progression and persistence of the disease [73,85,88]. Thus, psoriasis seems to arise in genetically predisposed individuals with an abnormal innate and adaptive immune response to environmental factors that results in a hyperproliferation of KCs, but the exact mechanisms behind these are not completely understood [89,90]. New evidence suggests that in addition to the model of immune-pathogenesis of psoriasis, other components such as neuropeptides released by the cutaneous nervous system and the skin microbiome may also be mediators in the process [73]. It has also been reported that de novo GC synthesis and GC receptor expression are dysfunctional in psoriatic KCs and this can also contribute to psoriasis pathogenesis since GC synthesis is essential in controlling skin inflammatory processes [91].

#### 3.2.1. Genetic Basis of Psoriasis

There is strong evidence supporting a genetic basis for psoriasis. The familial recurrence of psoriasis is well known and disease concordance is higher in monozygotic twins than in dizygotic twins [92]. Additionally, recent epidemiological studies have also confirmed that there is a genetic predisposition for psoriasis, with more than 20% of patients having a positive family history [93].

More than 80 psoriasis susceptibility loci have been identified to date [94]. In the human genome, 15 psoriasis-susceptible regions (PSORS) have been recognized through linkage analysis, with PSORS1 being the major genetic determinant of this disease [95,96]. This locus possesses 15 genes which are strongly associated with psoriasis [94]. The most important seems to be the HLA (human leukocyte antigen), as revealed by haplotype analysis, genome-wide association studies and analysis of single-nucleotide polymorphism [97]. Gene ontology and pathway enrichment analysis studies have recently shown that the genes that have been identified as having a possible connection to the pathogenesis of psoriasis cluster to a small number of immune pathways, such as antigen presentation, innate antiviral signaling and Th17 cell activation [95,96]. Ran et al. [94] grouped these genes into three main pathways: skin barrier function, innate immune response and acquired immune response.

#### 3.2.2. Neuroimmune Basis of Psoriasis

The central mechanisms behind psoriasis pathogenesis involve the crosstalk between IIS and AIS and chronic interactions between these and resident skin cells, mainly hyper-proliferative, dysfunctional KCs and infiltrating activated immune cells, largely composed of CD4+ and CD8+ T cells [12,26,86,98]. The cytokines TNF-α, IFN-γ and the IL-23/Th17 axis play predominant signaling roles in the process [13]. Hence, psoriasis can be considered an autoimmune disease with an (auto)inflammatory background, with both mechanisms overlapping, and the clinical presentation resulting from a balance between the two. Although both IIS and AIS contribute to the pathogenesis of psoriasis, there is evidence that chronic plaque psoriasis is mainly caused by an activation of the AIS, while generalized pustular psoriasis mainly involves the IIS and autoinflammatory responses [75].

Several cells and molecules play a mediator role in psoriasis, as summarized in Table 2, such as innate immunocytes, adaptive T cells, KCs and cytokines.

Initially, psoriasis was characterized as a Th1-driven disease because psoriatic lesions were related to an increase of Th1-type proinflammatory cytokines in relation to Th2-type anti-inflammatory cytokines. The discovery of a novel Th17 cell subset characterized by the production of IL-17, a highly proinflammatory cytokine that induces severe autoimmunity, shifted the model of psoriasis pathogenesis and conferred a central role to this type of CD4+ T cell [26,87,99]. In fact, while healthy human skin contains just a few Th17 cells, their concentration is highly increased in psoriasis and other inflammatory disorders [100]. The IL-17 family of cytokines consists of 6 proteins (IL-17A to IL-17F), with IL-17A and IL-17F being mainly produced by Th17 cells [101].

Recent research also shows a fundamental role for peptide and nonprotein lipid autoantigens in psoriasis, reinforcing the hypothesis of an autoimmune disease. Keratinocytes generate AMPs that complex with self-nucleic acid released from neutrophils and damaged KCs [100] and that function as autoantigens [102]. Three major classes of AMPs have been found in psoriatic skin: the cathelicidin LL37, human beta-defensins (hBD)1, hBD2 and hBD-3, and S100 proteins such as psoriasin (S100A7) [103]. Two-thirds of patients with moderate-to-severe plaque psoriasis have CD4+ and/or CD8+ T cells specific for LL37, which is also overexpressed in psoriatic skin [102]. Additionally, epidermal melanocytes, but also epidermal KCs, perivascular dermal cells and blood vessels, generate ADAMTSL5, a secreted zinc metalloprotease-related protein that regulates components of the extracellular matrix and can also act as an autoantigen [104,105].

In pre-psoriatic skin, injured KCs activated by trigger factors produce AMPs, IL-1 family cytokines and chemokines that act as chemoattractants for infiltrating LCs, pDCs, mDCs, neutrophils, MCs and macrophages [15,85,106]. The attracted pDCs initiate psoriasis inflammation [107] and are activated by the AMP–nucleic-acid complexes. This suggests that it is the overexpression of the AMPs by psoriatic KCs that breaks tolerance of pDCs to self-nucleic acids, promoting autoimmunity and providing the link between pDCs and KCs in the pathogenesis of psoriasis [98,103]. The complexes between AMPs and self-nucleic acids activate pDCs to produce IFN type I which, in turn, induce the functional maturation of mDCs, leading to the overproduction of TNF-α and IL-6 [31,32,98]. The pathogenicity of IFN-γ produced by early migrating pDCs is evidenced by the fact that psoriasis is exacerbated in patients being treated with this IFN for unrelated diseases [108]. Keratinocytes are also able to activate mDCs to produce IL-1β and IL-6 via IL-36 cytokines [109], and activate macrophages via overexpressed LL37, further promoting inflammation [85]. Furthermore, the LL37 produced by KCs also regulates the expression of cytokines of the IL-1 family by KCs themselves, and induces chemokine production in these cells, which participate in the recruitment of neutrophils in the early stages of psoriasis [110].

Activated pDCs and mDCs are essential to initiate the AIS response, but KCs are also involved since they trigger and condition DC responses [85]. The LL37 produced by KCs is recognized as an autoantigen by CD4+ and CD8+ T cells with a typical psoriatic receptor profile [102], while ADAMTSL5 only activates the latter [111]. Activated DCs produce IL-1β, IL-6, TGF-β1, IL-12, IL-18, IL-23 and TNF-α, mediators that are found in psoriatic plaque infiltrates [26,71,73,99], and that stimulate and maintain the differentiation of naïve T cells as discussed previously (Figure 2). The stimulation and differentiation of Th17 and Th22 cells (in the early stages) and Th1 (mainly during the chronic phase) occur via two main pathways: the IL-23/Th17 axis (also called IL-23/IL-17/IL-22 axis) and the TNF-α pathway [13,99]. The IL23/Th1 axis is characterized by high level production of IL-23 by DCs and KCs, and an increased number of Th17 cells [112]. Activated Th17 cells produce IL-17A, IL-17F and IL-22, which induce KC proliferation. Reciprocally, KCs further stimulate the production of IL-17 by Th17 in an IL-36-dependent manner, creating a positive feedback loop and making IL-36 cytokines important amplifiers of Th17 signaling [113]. All these processes result in an imbalance between Th17 and Treg cells, which promotes inflammation [114]. Interestingly, in psoriatic skin, IL-17 is not only produced by CD4+ T cells but also by epidermal CD8+ T cells and innate immune cells such as neutrophils, MCs and macrophages [115,116]. Similarly, IL-22 is not only produced by Th17 but also by MCs [116] and Th22 cells [117]. The IL-22 receptor in skin is only expressed in KCs; thus, this cytokine has an important but limited role in psoriasis when compared to IL-17. IL-22 promotes the release of chemokines and AMPs from KCs, inducing their proliferation and differentiation [85]. The TNF-α pathway also plays a central role in psoriasis, amplifying inflammation in several distinct ways. TNF-α is produced by a wide range of cells (macrophages, lymphocytes, KCs, endothelial cells), acts on several other cells and induces secondary mediators that have been implicated in psoriatic disease [13]. High levels of TNF and its receptors, TNFR1 and TNFR2, have been detected in psoriatic lesions, including in Th17 cells and KCs. TNF-α produced by DCs binds to receptors in Th17 cells, further activating them and leading to increased IL-17 production. Furthermore, it can also synergize with IL-17 to increase neutrophil recruitment [118].

**Figure 4:**
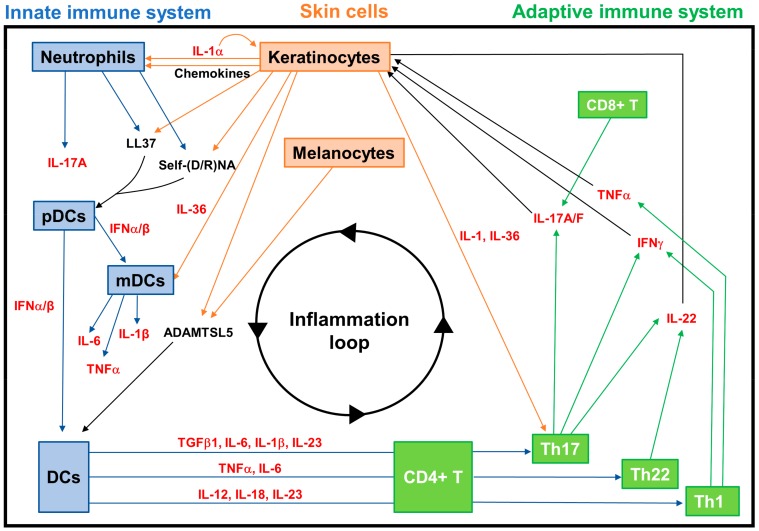
The inflammation loop at the core of psoriasis pathogenesis. The pathogenesis of psoriasis involves an interplay between innate immune cells (pDCs, mDCs, neutrophils, etc.), skin cells (keratinocytes, melanocytes, etc.) and adaptive immune cells (CD4+ and CD8+ T cells, Th1, Th17, Th22, Treg, etc.). The crosstalk between these cells is mediated by molecules such as antimicrobial peptides LL37 and ADAMTSL5, produced by keratinocytes and/or melanocytes, which act like autoantigens. Additionally, several cytokines, shown in red, and chemokines are fundamental in the process. DCs, dendritic cells; pDCs, plasmacytoid dendritic cells; mDCs, myeloid dendritic cells; IL, interleukin; TNF, tumor necrosis factor; IFN, interferon; TGF, transforming growth factor.

During the late/chronic phase of psoriasis, the close interactions between KCs, epidermal epithelial cells and innate and adaptive immune cells further amplify the inflammation cycle, sustaining the persistence of psoriasis [25]. In this phase, the T-cell infiltrate and DC subpopulations present in lesional areas mainly express cytokines IFN-γ, TNF-α, IL-17 and IL-22, which induce KCs to further overexpress inflammatory mediators. This causes the chronic skin inflammation that is responsible for the proliferation of KCs and endothelial cells, neo-angiogenesis and infiltration of dendritic cells [71,85,119]. The inflammatory mediators produced by KCs during this late phase contribute to the abnormal proliferation and differentiation of the epidermis, resulting in a self-amplifying loop which acts back on DCs, neutrophils and T cells, perpetuating the inflammation processes (Figure 4) [85].

#### 3.2.3. The Role of Serotonin in Psoriasis

Serotonin mediates the bidirectional interactions between skin and the neuroendocrine system, as discussed in Section 2.3 [8]. Thus, it is not surprising that several studies suggested a role for this neurotransmitter in the pathogenesis of psoriasis. Additionally, 5-HT is also a main factor in depression, which is one of the main comorbidities of psoriasis. In fact, it has been reported that psoriasis is associated with a 39% increase in depression and 31% increase in anxiety [120], while a recent population-based cohort study revealed that the highest risk for depression occurred for mid-aged patients with severe forms of psoriasis and patients with a prior history of depression [121]. Moreover, the cytokine theory of depression suggests that depression and other mental disorders have hormonal and biochemical components, and might involve inflammation processes [122]. This was, for example, supported by studies of 5-HTT and cytokines expression in blood of depressed patients compared to healthy controls, with results showing an increase in the mRNA levels of SERT, IL-1β, IL-6 and TNF-α [123]. Furthermore, it has been reported that selective serotonin reuptake inhibitors (SSRIs) influence the topical symptoms of psoriasis [124,125,126]. All these results reveal an association between psoriasis–inflammation–serotonin. The role of the serotonergic system in skin and its interactions with skin immunocytes and non-immunocytes were already reviewed in Section 2.3.3. Here, the relationship between the serotonergic system and psoriasis will be discussed (summarized in Table 3).

The presence of 5-HT in skin was revealed by immunohistochemical studies, and the comparison between normal and psoriatic skin revealed a possible role for 5-HT in psoriasis. Huang et al. [127] studied the expression of 5-HT in skin lesions of psoriatic patients. The immunohistochemistry results revealed the expression of 5-HT in psoriatic lesions, but not on normal skin cells. The expression was significantly higher in epithelial and adnexal structures in the progressive stage of psoriasis, but weak in the static stage, while no increase was observed in MCs or LCs of the same samples. The authors suggested that the role of 5-HT in inflammation might involve the stimulation of KCs proliferation, as well as a direct influence over CD4+ T-lymphocytes. Younes and Bakry [128] performed similar studies in skin biopsies from patients with chronic plaque psoriasis and compared the results with the ones obtained for healthy skin subjects. Just like in Huang’s study [127], 5-HT expression was significantly higher in psoriatic compared to normal skin, with 5-HT expressed in basal and suprabasal skin layers of psoriatic patients. The authors hypothesized that the expression in the suprabasal layer may be related to the action of 5-HT in KC differentiation.

The expression of 5-HT receptors is also significantly altered in psoriatic skin. Nordlind et al. [129] used immunohistochemical methods to study the differential expression of 5-HT1AR, 5-HT2AR and 5-HT3R in the epithelial and annex structures of psoriatic skin, compared to normal skin. The results showed a lower expression for 5-HT1AR and higher expression for 5-HT2AR in psoriatic dermis compared to normal skin, whereas 5-HT3R was detected in the basal epidermis layer of noninvolved psoriatic skin. These differential expressions of 5-HT1AR and 5-HT2AR are in agreement with the functions of these receptors: 5-HT1AR is known to be involved in the inhibition of inflammation, while the type 2 receptors are proinflammatory, being involved in the 5-HT-mediated recruitment of CD4+ T-lymphocytes to inflammatory foci [6,62]. Additionally, experiments using a 5-HT3R antagonist (tropisetron), suggested that this receptor might mediate the pruritus developed in response to iontophoretically applied 5-HT in human skin, thus being expressed on sensory nerve endings, as discussed in Section 2.3.3. [68]. In situ immunochemical studies using human skin biopsies also revealed the expression of 5-HT3R in the basal epidermal layer of both normal and eczematous skin [6,57]. More recently, Morita et al. [130] have shown that 5-HT7R is also a key mediator of serotonergic acute and chronic itch, and this receptor is also present in skin, as previously shown by Slominski et al. [56]. All these results suggest that these receptors may be possible targets for anti-inflammatory therapies in psoriasis [129].

The 5-HT transporter (SERT) is also differentially expressed in the skin of psoriasis patients compared to healthy skin [40]. Regarding psoriatic skin, the number of SERT-positive DCs in the epidermis was significantly higher than in normal skin, as was the number of epidermal inflammatory cells immunostained for SERT. The fact that SERT immunostaining co-localized with caspase-3 suggested that the 5-HT transporter may be involved in regulating apoptosis in psoriasis inflammatory cells. Furthermore, it was observed that the majority of SERT-positive cells in the epidermis were DCs, while in the dermis mostly MCs and lymphocytes were SERT-positive [6,40]. The increased SERT expression in epidermal inflammatory cells correlated to psoriasis severity and chronic stress, further supporting a role for the serotonergic system in the pathogenesis of this disease [6]. Thus, SERT may play a role in the regulation of inflammatory cells apoptosis, and it should also be considered as a possible target for antipsoriatic therapy [40].

## 4. Conclusions and Future Directions

This review focuses on the skin as a complex organ, with immune, endocrine and neurological functions similar to the brain. Serotonin, the classical central nervous system neurotransmitter with a well-known role in depression, also plays a fundamental role in skin homeostasis. The skin immune and serotonergic systems are connected: several skin immunocytes and non-immunocytes synthesize, transport and/or are regulated by 5-HT via multiple membrane serotonin receptors; on the other hand, cytokines produced by the immune system and/or skin cells also regulate the action of the serotonergic system.

The brain–skin connection helps explain why psychological stress can have effects on skin, including the development or exacerbation of skin diseases such as psoriasis, atopic dermatitis and eczema. Psoriasis is a disease with a very complex pathogenesis involving genetic factors, skin cells, innate and adaptive immune cells and a network of signaling molecules including autoantigens, cytokines and chemokines. The results that have been reported include in vitro assays, ex vivo assays, in vivo assays using animal models, in vivo assays in humans, clinical trials, cell biology, molecular biology and, more recently, genome-wide assays at the level of transcriptomics [131], proteomics [132], metabolomics [133], as well as their multi-omics integration and in silico predictions via modeling approaches [134]. This systems biology approach has been applied to help unravel the pathological mechanisms in human diseases, such as Alzheimer’s [135], coronary artery disease [136], cardiovascular disease, type 2 diabetes [137] and cancer [138], among others. We believe that a similar approach will prove extremely useful to better understand the pathogenesis of psoriasis, making it easier to find new therapeutic strategies for this disease. Data obtained from large-scale studies combined with individual genetic profiles will be extremely useful to define prognosis and develop personalized medicine for psoriasis patients [139].

## Figures and Tables

**Table 1:** The serotonergic system in human immune and skin cells. Note that some of these studies detected the gene expression (mRNA levels) which does not mean that the mRNA is translated into protein.

	Cells	5-HT Metabolism and Transport	5-HT Receptors
**Innate immune system**	Dendritic cells	SERT [40]	5-HT1BR, 5-HT1ER, 5-HT2AR, 5-HT2BR, 5-HT3R, 5-HT4R, 5-HT7R [41]
	Mast cells	TPH1 [42]	5-HT1AR (main receptor in human and mice); human MCs also express mRNA for receptors 5-HT1B, 5-HT1E, 5-HT2A, 5-HT2B, 5-HT2C, 5-HT3, 5-HT4 and 5-HT7 [43]
	Macrophages	SERT [44]	5-HT1AR, 5-HT2AR, 5-HT2BR, 5-HT2CR, 5-HT3R, 5-HT7R [45,46]
**Adaptive immune system**	T cells	TPH1 [47], MAO, SERT [48]	5-HT1AR [49], 5-HT1BR [47], 5-HT2BR [50]; 5-HT3AR (in activated CD4^+^ Th) [51,52]
	B cells	SERT [53]	5-HT1AR [54], 5-HT3AR [55]
**Skin cells**	Keratinocytes	TPH1 [21]	5-HT1AR, 5HT1BR, 5-HT2AR, 5-HT2BR, 5-HT2CR, 5-HT7R [56], 5-HT3R [57]
	Melanocytes	TPH1 [21]	5-HT1AR, 5-HT1BR, 5-HT2AR, 5-HT2BR, 5-HT2CR, 5-HT7R [56]

**Table 2:** Cells and signaling molecules that play a central role on the pathogenesis of psoriasis.

	Type	Name	Action
**Cells**	Innate immunocytes	Antigen-presenting cells (APCs)	Include Langerhans cells (LCs) in the epidermis, dendritic cells (DCs) and macrophages in the dermis. They present the antigen to T cells which recognize it via a T-cell receptor and become activated.
		Mast cells (MCs)	Granulocyte cells that contain histamine and are involved in allergy reactions, but can also activate and recruit immune-competent cells; can be induced to become APCs.
		Neutrophils	Most common type of leukocytes in the blood, early markers of inflammation; can be induced to become APCs.
		Natural killer (NK) cells	Cytotoxic lymphocytes which do not require activation to kill cells that do not have markers (antigens).
	Adaptive immunocytes	Conventional T cells	T lymphocytes: CD4^+^ Th cells, cytotoxic CD8^+^ T cells, which can become long-term resident memory T cells (TRMs). Depending on the cytokine milieu, naïve CD4^+^ T cells differentiate into Th1, Th2, Th17, T_reg_, etc.
		NK T cells	Innate-like T lymphocytes that share surface markers and functional characteristics with conventional T cells and NK cells.
		B cells	B lymphocytes that express B cell receptors on their cell membrane that can bind to an antigen initiating the antibody production (humoral immunity).
	Non-immunocytes	Keratinocytes (KCs)	Epidermal cells responsible for the protective barrier function of the skin. Upon invasion of the upper layer of the epidermis, they produce proinflammatory signals (IL-1 family cytokines, AMPs, chemokines) which mediate their crosstalk with innate and adaptive immune cells.
		Melanocytes	Epidermal cells that generate the autoantigen ADAMTS-like protein 5 (ADAMTSL5).
**Signaling molecules**	Cytokines	Interleukins (IL)	Th1-type proinflammatory ILs: IL-2; Th2-type anti-inflammatory interleukins: IL-4, IL-5, IL-6, IL-9, IL-10, IL-13; Th17-type pro-inflammatory IL-17; IL-1-type pro-inflammatory IL-36; Th22-type pro-inflammatory IL-22; IL-12 family pro-inflammatory IL-23.
		Interferon (IFN)-α	Pro-inflammatory cytokine mainly produced by plasmacytoid dendritic cells (pDCs) during the early phases of psoriasis.
		Interferon (IFN)-γ	Th1-type proinflammatory cytokines; mainly produced by NK and NK T cells, CD4^+^ Th1 and Th17 cells, and CD8^+^ cytotoxic T cells.
		Tumor necrosis factor (TNF)-α	Proinflammatory cytokine produced by macrophages, monocytes, endothelial cells, neutrophils and activated lymphocytes.
		Tumor necrosis factor (TNF)-β	Proinflammatory cytokine mainly produced by KCs.
	Chemokines	CXCL9, CXCL10, CXCL11, CCL20, chemerin, etc.	Small cytokines which act as mediators of innate immune cells chemotaxis, may be produced by KCs and leukocytes.
	Autoantigens	LL37	Produced by KCs, it complexes with self-nucleic acids derived from damaged KCs and neutrophils, and acts as an autoantigen, activating pDCs via TLRs, and also being recognized by CD4+ and CD8+ T cells.
		ADAMTSL5	Produced by melanocytes and KCs, it is able to activate DCs and is recognized as an autoantigen by CD8+ T cells.

**Table 3:** Changes in the serotonergic system in psoriatic skin.

		Changes in Psoriatic Skin
**Serotonin**	5-HT	Expression increased in epithelial and adnexal structures of psoriatic skin [127].
		Expression increased in basal and suprabasal skin layers in psoriatic skin [128].
**5-HT receptors**	5-HT1AR	Lower expression in psoriatic dermis [129].
	5-HT2AR	Increased expression in psoriatic dermis [129].
	5-HT3R	Increased expression in the basal epidermis of noninvolved psoriatic skin [129].
		Increased expression in sensory nerve endings [68].
**5-HT transporter**	SERT	Increased expression in DCs and other inflammatory cells in the epidermis [6,40].
		Increased expression in MCs and lymphocytes in the dermis [6,40].
